# Draft Genome of *Shewanella frigidimarina* Ag06-30, a Marine Bacterium Isolated from Potter Peninsula, King George Island, Antarctica

**DOI:** 10.1128/genomeA.00289-16

**Published:** 2016-05-05

**Authors:** Gisela Parmeciano Di Noto, Susana C. Vázquez, Walter P. MacCormack, Andrés Iriarte, Cecilia Quiroga

**Affiliations:** a Instituto de Investigaciones en Microbiología y Parasitología Médica, Universidad de Buenos Aires, Consejo Nacional de Investigaciones Científicas y Tecnológicas (IMPaM, UBA-CONICET), Facultad de Medicina, Buenos Aires, Argentina; b Nanobiotec, Facultad de Famarcia y Bioquímica, UBA-CONICET, Buenos Aires, Argentina; c Departamento de Microbiología Ambiental, Instituto Antártico Argentino, Buenos Aires, Argentina; d Departamento de Desarrollo Biotecnológico, Instituto de Higiene, Facultad de Medicina, Universidad de la República, Montevideo, Uruguay; e Departamento de Bioquímica y Genómica Microbianas & Departamento de Genómica, IIBCE, Montevideo, Uruguay

## Abstract

We present the draft genome of *Shewanella frigidimarina* Ag06-30, a marine bacterium from King George Island, Antarctica, which encodes the carbapenemase SFP-1. The assembly contains 4,799,218 bp (G+C content 41.24%). This strain harbors several mobile genetic elements that provide insight into lateral gene transfer and bacterial plasticity and evolution.

## GENOME ANNOUNCEMENT

*Shewanella* spp. are Gram-negative bacteria with highly versatile respiration systems that thrive in aquatic niches under different environmental conditions; however, some species are also opportunistic pathogens (1, 2). *Shewanella* spp. have very plastic genomes as a result of the presence of several mobile genetic elements (MGE), which contribute strongly to bacterial evolution and adaptation (3). Moreover, some *Shewanella* spp. encode OXA-48-type emerging carbapenemases, which have been transferred to multidrug resistant *Enterobacteriaceae* (4, 5). Analyses of *Shewanella* genomes provide useful information on the evolution and adaptation of this organism to many niches and their participation in MGE and antimicrobial resistance gene transfer. Here, we report the draft genome of *Shewanella frigidimarina* strain Ag06-30, which was isolated from intertidal seawater on a resting area of southern elephant seals (*Mirounga leonina*) on Potter Peninsula, King George Island (Isla 25 de Mayo), South Shetland Islands, Antarctica (62°15′22″ S, 58°37′24″ W). Bacteria were grown on mineral basal media with 1.6% agar at 4°C. A small cream-colored colony was purified and stored at −80°C. 16s rDNA sequence analysis identified this isolate as *Shewanella* sp*.* Ag06-30. Total DNA extracted using a Wizard genomic DNA purification kit (Promega) was sequenced on the Illumina MiSeq at the Argentine Genomic Technology Consortium. A total of 2,286,950 high-quality reads were obtained and filtered to remove adapters using Scythe (https://github.com/vsbuffalo/scythe). Assembly of 99.0% of the total generated reads (average length: 282 bp; paired-read span: 580 bp) resulted in a mean nucleotide coverage of 127.7 (k-mer coverage of 74.4). A draft genome was generated by *de novo* assembly by means of SPAdes v3.6.2 (6), using a preassembly approach with Velvet 1.2.10 (7). The draft genome is composed of a total of 4,799,218 bp, distributed in 127 contigs with an *N*_50_ value of 156,173 and a largest contig size of 940,645 bp. The genome showed a G+C content of 41.24%. Contigs were submitted to the Rapid Annotation using Subsystem Technology server (8), which identified 4,265 open reading frames (ORFs). The complete sequence was submitted to GenBank, where final annotation was implemented using the NCBI Prokaryotic Annotation Pipeline (9).

Assembled contigs longer than 500 pb were used to estimate the average nucleotide identity (ANI) between *Shewanella* sp. Ag06-30 and the complete genomes of *Shewanella* spp. available in GenBank. Two-way ANI (reciprocal best hits based comparison) analysis revealed that strain Ag06-30 was closest to *S. frigidimarina* NCIMB400 (ANI: 96.87% [SD: 2.84%]), known for its ability to reduce Fe^3+^ (10). Comparative genome analysis between both strains showed that *S. frigidimarina* Ag06-30 has all metabolic pathways described for *S. frigidimarina* NCIMB400 and several unique regions (10, 11). Mobilome analysis showed the presence of one genomic island; 14 prophage-related proteins; 16 insertion sequences from families IS*1595*, IS*66*, and IS*4*; an integron integrase; and a class C-attC group II intron (12–15). This strain did not harbor plasmids (16). Strain Ag06-30 also encoded carbapenemase SFB-1 (17). *S. frigidimarina* Ag06-30 genome analysis provides novel insight on the plasticity and evolution of the *Shewanella* genus.

### Nucleotide sequence accession number.

The genome sequence of *Shewanella frigidimarina* Ag06-30 has been deposited in the GenBank database under the accession number LRDC00000000.
